# Accelerating Elastic Property Prediction in Fe-C Alloys through Coupling of Molecular Dynamics and Machine Learning

**DOI:** 10.3390/ma17030601

**Published:** 2024-01-26

**Authors:** Sandesh Risal, Navdeep Singh, Yan Yao, Li Sun, Samprash Risal, Weihang Zhu

**Affiliations:** 1 Department of Mechanical Engineering, University of Houston, Houston, TX 77204, USA; srisal@cougarnet.uh.edu (S.R.); lsun3@central.uh.edu (L.S.); 2 Department of Mechanical Engineering, School of Engineering and Computer Science, University of the Pacific, Stockton, CA 95211, USA; 3 Materials Science and Engineering Program, University of Houston, Houston, TX 77204, USA; yyao4@central.uh.edu (Y.Y.); srisal2@cougarnet.uh.edu (S.R.); 4 Department of Electrical and Computer Engineering, University of Houston, Houston, TX 77204, USA; 5 Department of Engineering Technology, University of Houston, Houston, TX 77204, USA

**Keywords:** machine learning, molecular dynamics, density functional theory, MEAMfit, RF-MEAM, elastic properties, mechanical properties

## Abstract

The scarcity of high-quality data presents a major challenge to the prediction of material properties using machine learning (ML) models. Obtaining material property data from experiments is economically cost-prohibitive, if not impossible. In this work, we address this challenge by generating an extensive material property dataset comprising thousands of data points pertaining to the elastic properties of Fe-C alloys. The data were generated using molecular dynamic (MD) calculations utilizing reference-free Modified embedded atom method (RF-MEAM) interatomic potential. This potential was developed by fitting atomic structure-dependent energies, forces, and stress tensors evaluated at ground state and finite temperatures using ab-initio. Various ML algorithms were subsequently trained and deployed to predict elastic properties. In addition to individual algorithms, super learner (SL), an ensemble ML technique, was incorporated to refine predictions further. The input parameters comprised the alloy’s composition, crystal structure, interstitial sites, lattice parameters, and temperature. The target properties were the bulk modulus and shear modulus. Two distinct prediction approaches were undertaken: employing individual models for each property prediction and simultaneously predicting both properties using a single integrated model, enabling a comparative analysis. The efficiency of these models was assessed through rigorous evaluation using a range of accuracy metrics. This work showcases the synergistic power of MD simulations and ML techniques for accelerating the prediction of elastic properties in alloys.

## 1. Introduction

The prediction of material properties using computational tools can serve as a crucial guide to the experimental material design process [1]. Computational methods like density functional theory (DFT) and molecular dynamics (MD) are able to compute material properties accurately, allowing researchers to gain meaningful and prompt insights into the material properties of interest [2]. However, their applicability is constrained by limitations in length (restricted to a few nanometers [3,4]) and time scale (limited to a few nanoseconds [5,6,7]) that can be explored efficiently and effectively.

In recent years, machine learning (ML) has gained popularity for predicting material properties as evidenced by notable studies [8,9,10,11,12]. For ML, the data availability is a significant hurdle. The integration of ML with these multi-level computational material tools addresses the individual limitations of each method, accelerating the novel material discovery. This synergy solves the data scarcity in ML by generating a plethora of data using DFT and MD and the time limitation of the computational tools by using ML that further expedites the materials exploration process.

Numerous studies have harnessed ML models to predict mechanical properties, including tensile strength, yield strength, hardness, and elongation, across various alloy systems. Here, we present a literature review that underscores the diverse applications of ML in predicting alloy mechanical properties and subsequently focus on its application in predicting steel properties.

The quest to predict the mechanical properties of alloys using machine learning has seen significant advancements across various alloy systems. Hu et al. [13] proposed a novel feature engineering method integrating chemical compositions and manufacturing processes, achieving high prediction accuracy for wrought aluminum alloys. Deng et al. [14] further refined this approach, employing a sequential minimal optimization algorithm to predict copper-aluminum alloys’ tensile strength and hardness. With the guidance of their model, they successfully developed an alloy meeting specific mechanical property targets.

Devi et al. [15] compared multiple ML models for predicting aluminum alloys’ tensile strength and hardness, demonstrating the efficacy of algorithms like K-nearest neighbors (KNN) and artificial neural networks (ANN) in this domain. Similarly, Xu et al. [16] employed ANN and support vector machine (SVM) models to predict properties in magnesium alloys, showcasing the potential for relating composition, processing, and mechanical properties.

Choyi [17] applied ML techniques to screen and identify promising alloy compositions for experimental testing in aluminum alloys. Ling [18] extended this approach by utilizing ensemble ML to predict mechanical properties and phase transformation temperatures for various alloy classes, enabling an inverse design framework for creating new alloys meeting specific specifications.

Lu et al. [19] focused on magnesium-rare earth alloys, utilizing SVR-based ML models to predict multiple mechanical properties with considerable accuracy. Tan et al. [20], employing a simpler ML model, provided a foundational tool for predicting mechanical properties in multi-principal element alloys based solely on composition data.

Zhang et al. [21] and Lee et al. [22] explored steel alloys, utilizing ML to predict tensile strength and yield strength while employing various algorithms and input parameters. Shen et al. [23] pioneered a physical metallurgy-guided ML approach, integrating equilibrium volume fraction and driving force for precipitation into their models for predicting hardness, leading to the design of ultrahigh-strength stainless steel with remarkable accuracy. Further extending these methodologies, Mahfouf [24], Gaffour et al. [25], Dutta et al. [26], and Pattanayak et al. [27] utilized a range of ML techniques such as artificial neural networks (ANN), genetic algorithms (GA), and hybrid models to model mechanical properties, optimize compositions, and predict mechanical performance across various steel alloys, showcasing the potential of these methods in alloy design and optimization.

A thorough review of the literature [13,14,15,16,17,18,19,20,21,22,23,24,25,26,27] above demonstrates that various ML algorithms such as LR, SVM/SVR, RF, ANN, KNN, and GPR are effective at making predictions. Notably, some studies have also explored ensemble techniques such as XGBoost, AdaBoost, and SL to enhance predictive accuracy. The majority of these studies utilized composition and process parameters as their primary inputs. Among the commonly employed process parameters were heat treatment temperature, heating duration, choice of cooling medium, and others. Hence, a broad spectrum of strategies have been applied in materials property prediction through ML methodologies.

In this work, we aim to demonstrate a framework to predict the elastic properties of iron-carbon systems using the synergistic effect of multiscale computational material tools (DFT and MD) and ML. MD calculations using the interatomic potential, produced by fitting on DFT-calculated data, generate the dataset, which is subsequently utilized to train the ML model for the expedited prediction of elastic properties.

## 2. Data and Methods

### 2.1. Potential Development

Potential development methodology and calculation details have been discussed in our previous publication [28]. Here, we briefly summarize the process to provide a complete picture of the framework that integrates the previous work with the ML model for elastic properties prediction.

The inter-atomic potential was developed by fitting the potential parameters to the forces, energies, and stress tensors of various ordered structures of Fe-C calculated by first principle calculations using Vienna Ab initio Simulation Package (VASP) [29] with the projector augmented wave (PAW) [30] pseudopotentials. The exchange-correlation function was calculated using the Perdew–Burke–Ernzerhof Generalized-Gradient Approximation (GGA-PBE) [31]. These precise data were fitted into 78 parameters of RF-MEAM potential [32] using a potential fitting code called MEAMfit [33]. Thus, the produced potential was used to perform MD calculation on LAMMPS [34] to reproduce the properties of the structures used during the fitting process. The potential was further verified by calculating elastic moduli for the disordered structures at various finite temperatures.

### 2.2. Data Collection/Calculation

The training data for ML were generated through MD simulations using the developed RF-MEAM potential. These calculations were performed on various ordered and disordered structures. The ordered structures used are B1, B3, and Cementite, whereas the disordered alloy structures used were Fe-C with fcc and bcc base structures, each with octahedral and tetrahedral interstitial carbon atoms. For these disordered alloys, the composition of carbon was varied. The carbon concentrations used were 1, 2, 3, 4, 5, 6, 8, 10, 12, 14, 16, 18, and 20 atomic percentage. These structures were subjected to temperatures from 0 K to 1200 K in 50 K increments and elastic properties were calculated at each temperature. In total, 1375 data points were obtained and used for this study.

The atomic structure is initially equilibrated at the desired temperature using the NPT ensemble, allowing the system to equilibrate under constant temperature and pressure conditions. The equilibrated structures are then subjected to positive and negative deformations (i.e., deformation applied along specific axes in two opposite directions) in six directions, corresponding to the Voigt deformation components. These deformations were applied to investigate the material’s response to mechanical stress. After each deformation, the structures were equilibrated again using the NVE ensemble. The resultant change in stress within the structure was measured after each deformation. These data were used to compute the elastic stiffness tensor, which characterizes the material’s response to mechanical deformation. Once we have the elastic stiffness tensors, Voigt equation

(1) $$B_{V}=\frac{1}{9}\left(c_{11}+c_{22}+c_{33}\right)+\frac{2}{9}\left(c_{12}+c_{13}+c_{23}\right),$$

and

(2) $$G_{V}=\frac{1}{15}\left(c_{11}+c_{22}+c_{33}-c_{12}-c_{13}-c_{23}\right)+\frac{1}{5}\left(c_{44}+c_{55}+c_{66}\right),$$

Reus equation

(3) $$B_{R}=\frac{1}{\left(s_{11}+s_{22}+s_{33}\right)+2\left(s_{12}+s_{13}+s_{23}\right)},$$

and

(4) $$G_{R}=\frac{1}{4\left(s_{11}+s_{22}+s_{33}\right)-4\left(s_{12}+s_{13}+s_{23}\right)+3\left(s_{44}+s_{55}+s_{66}\right)},$$

and Hill equation

(5) $$B_{H}=\frac{1}{2}\left(B_{R}+B_{H}\right)$$

and

(6) $$G_{H}=\frac{1}{2}\left(G_{R}+G_{H}\right)$$

are used to calculate the macro elastic constants like bulk modulus, and rigidity modulus. The Youngs modulus can be calculated using equation

(7) $$E_{H}=\frac{9B_{H}G_{H}}{3B_{H}+G_{H}}.$$

These three elastic constants are used as the target properties for our ML study.

### 2.3. Input Parameters

The input parameters for ML should be meticulously selected to correlate well with the output target properties. So, we initially did a simple visual analysis by plotting the target properties vs. the possible input parameters. The purpose of this visual test is to observe if there is any relationship between the input and the output no matter how complicated the relation is. The input parameters we used are temperature, composition, base lattice structure type, interstitial sites, Ordered or Disordered, and three lattice constants a, b, and c.

Next, feature importance is calculated to see the effect of each input feature on the target properties. We used an RF regressor to calculate and feature importance library from yellow-brick to visualize feature importance.

### 2.4. ML Algorithms

**Figure 1:**
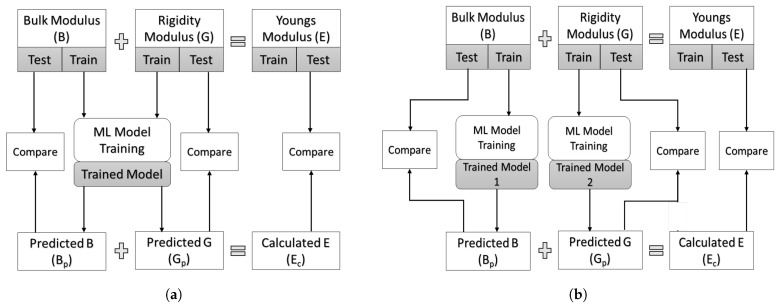
Two different approaches used for the properties prediction: (**a**) multivariate method, and (**b**) independent method.

Various ML algorithms were employed in this study to perform predictions using two distinct approaches, as shown in Figure 1. The first approach uses a multi-variate model to predict both the bulk and rigidity modulus and subsequently calculate Young’s modulus from these simultaneously predicted results. The second approach utilizes two separate models, each trained independently to predict their respective target properties, and then Young’s modulus is computed from the calculated bulk (B) and rigidity (G) modulus values.

This study employed a selection of individual algorithms known for their effectiveness in predicting material properties. Additionally, the ensemble algorithm named SL is utilized, which has demonstrated efficacy in combining the strengths of individual algorithms, making it particularly well-suited for small to medium-sized datasets, such as the one used in this study. The specific algorithms used in this study, along with the SL, are discussed below.

#### 2.4.1. Random Forest (RF)

RF is a bagging algorithm technique that combines multiple decision trees to determine the final result rather than relying on individual decision trees [35]. RF has numerous decision trees as base learning models. In RF, row and feature sampling are performed from the dataset creating sample datasets for every model. This part is called Bootstrap. Every decision tree has high variance, but when we combine them all in parallel, the resultant variance is low as each decision tree is perfectly trained on that particular sample data. Hence, the output does not depend on one decision tree but on multiple decision trees [36]. For a classification problem, the final output is taken using the majority voting classifier. Likewise, for a regression problem, the final output is the mean of all the outputs. This part is Aggregation [37].

#### 2.4.2. Extreme Gradient Boosting (XGBoost)

XGBoost is a ML method that combines the predictions of multiple weak Decision Tree (DT) models to produce a more robust prediction [38]. Initially, a model is built from the training data. Then, the second model is built to rectify the errors in the first model. The procedure is continued, and models are added until the complete training data set has been predicted or classified correctly or the maximum number of models has been added.

XGBoost, operating through a sequential process, constructs decision trees. All the independent variables fed into the decision tree are assigned weights, which play an essential role in XGBoost. The weight of variables predicted wrong by the tree is increased, and these variables are then fed to the second decision tree. These individual classifiers/predictors then ensemble to give a strong and more precise model. It can work on regression, classification, ranking, and user-defined prediction problems [39].

#### 2.4.3. Support Vector Machine (SVM)

SVM is a robust supervised ML algorithm used for both classification and regression tasks [40]. SVM seeks the optimal hyperplane that best separates data points belonging to different classes while maximizing the margin between the classes. Support vectors, which are the data points closest to the decision boundary, are crucial in determining the hyperplane [41]. SVM works well for both linear and non-linear classification problems using kernel functions such as linear, polynomial, sigmoid kernels, and radial basis functions (RBF).

SVM is known for its ability to perform well in high-dimensional spaces and its effectiveness in cases where the data are not linearly separable. It is widely used in applications like image classification, text classification, and bioinformatics [42].

#### 2.4.4. K-Nearest Neighbor (KNN)

KNN is a simple yet effective supervised ML algorithm used for classification and regression tasks [43]. KNN operates on the principle of identifying the ‘K’ nearest data points within the training set to a new, unseen data point and making predictions based on the majority class (for classification) or averaging (for regression) among those ‘K’ neighbors [44]. The choice of K, the number of nearest neighbors, is a critical parameter that affects the algorithm’s performance.

KNN is a non-parametric and instance-based algorithm, meaning it does not make explicit assumptions about the underlying data distribution. It works well for linear and non-linear data and is particularly useful for problems with complex or irregular decision boundaries [45]. However, its computational complexity increases with the size of the training dataset, making it less suitable for large-scale applications [46].

#### 2.4.5. MultiLayer Perceptron (MLP)

MLP, the most widely used neural network structure, consists of multiple layers of three types, i.e., input layer, output layer, and multiple hidden layers. The input layer receives the input signals to be processed. The output layer performs the required tasks, such as prediction and classification [47]. The hidden layers, placed in between the input and output layers, are the true computational engine of the MLP. Data passes in a forward path from the input to the output layer in MLP, equivalent to a network in feed-forward. The backpropagation learning technique is used to train all the nodes in the MLP. MLPs can fix issues that are not linearly separable and are structured to approximate every continuous function [48].

As with all neural networks, the dimension of the input vector dictates the number of neurons in the input layer, while the number of classes to be learned dictates the number of neurons in the output layer. The number of chosen hidden layers and neurons in each layer must be empirically determined. As a rule of thumb, the neurons in the hidden layers are chosen as a fraction of those in the input layer. However, there is a trade-off regarding the number of neurons: Too many neurons produce over-training; too few neurons affect generalization capabilities [49]. In this study, three hidden layers are used and the number of neurons in each layer is determined by the hyperparameter optimization.

#### 2.4.6. Gaussian Process Regression (GPR)

GPR is a probabilistic ML algorithm that, unlike many other ML models, allows for the prediction of the underlying uncertainties in its predictions [50]. GPR’s adaptability is another standout feature; it excels at modeling complex relationships in data without requiring rigid functional forms or predefined features. This adaptability is highly beneficial when dealing with noisy or limited datasets, enabling GPR to capture intricate patterns effectively [51].

However, GPR’s suitability depends on the specific characteristics of the problem. While it excels in scenarios with small datasets and noisy observations, it performs optimally in low-dimensional problems, stable design spaces, and moderately sized datasets [52]. Despite this specialization, GPR remains an invaluable tool in various domains, where its ability to provide probabilistic predictions and account for uncertainties makes it a vital asset in critical decision-making processes. Whether in geostatistics [53], finance [54], robotics [55], or materials [51], GPR’s probabilistic nature ensures that decision-makers receive accurate predictions and gain crucial insights into the confidence level associated with those predictions, ultimately enhancing the quality of informed decisions.

#### 2.4.7. Super Learner (SL)

SL is an ensemble ML approach designed to improve predictive accuracy and robustness by combining multiple base models or learning algorithms, encompassing a wide array of ML algorithms, from decision trees to neural networks. The concept aims to find the optimal combination of models to achieve the best possible prediction performance [56]. These single algorithm (base) models are trained on the same dataset, and their predictions are recorded as “meta-features”. Then, a meta-learner is introduced to the mix, which learns how to combine or weight the base models’ predictions optimally. This meta-learner, which can be as simple as linear regression or more complex like a neural network, aims to extract the best predictive insights from the collective knowledge of the base models.

SL’s appeal lies in its ability to deliver superior predictive performance consistently. It often outperforms any single base model in isolation by leveraging various models and learning algorithms. Furthermore, it enhances the robustness of predictions by reducing the risk of overfitting, as it avoids relying too heavily on the peculiarities of any one model [57]. Its flexibility is also noteworthy, as it can adapt to various data types and domains, offering a versatile solution for diverse ML challenges. Overall, the SL serves as a valuable tool for data scientists and ML practitioners seeking to maximize prediction accuracy and automate the model selection process while benefiting from the collective intelligence of multiple models [58].

## 3. Results and Discussion

### 3.1. Potential Verification

The developed interatomic potentials (i.e., different potentials fitted on various combinations of structures) were used to reproduce the elastic properties of various ordered and disordered alloys of Fe-C.

At first, energy as a function of volume was calculated for the ordered structures and fitted to the Birch–Murnaghan equation of state to obtain the bulk modulus of elasticity and its pressure derivative. These outputs were compared to experimental and DFT results in order to assess the accuracy and reliability of the potentials, allowing us to identify the best potential. Upon comparing the results from the potentials fitted on various distinct dataset combinations, it was found that the potential fitted on the dataset containing B1 and Cementite reproduced most of the alloy results faithfully. The RF-MEAM interatomic potential, referred to hereafter, is the potential fitted on the B1 and Cementite datasets. Table 1 details the results for equilibrium volume ($V_{0}$), bulk modulus ($B_{0}$), and pressure derivative of bulk modulus (${B_{0}}^{\prime }$) produced by our best DFT potential in comparison to the experimental and DFT results from the literature. From the table, it is apparent that the results from our best potential are in very good agreement with the literature.

**Figure 2:**
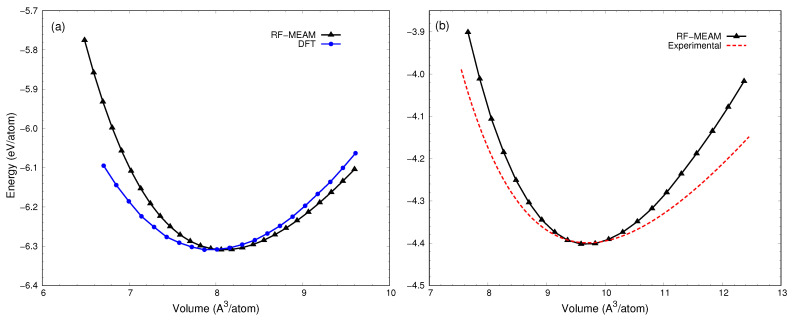
Comparison of energy vs. volume curves for (**a**) B1, and (**b**) Cementite with experimental and DFT results. Both DFT and experimental results used for the comparison are from Lalitha et al. [61].

Figure 2 compares the cohesive energy versus volume curve for (a) B1 and (b) Cementite with the DFT and experimental results, respectively, as reported by Lalitha et al. [61]. As seen in the figure, our calculated curves are in good agreement with the reported literature, with the equilibrium volume deviation of 2.4%, and 1.2% for B1, and Cementite, respectively.

**Figure 3:**
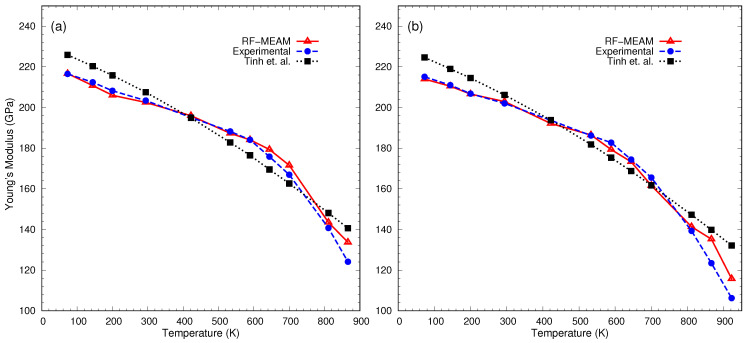
Young’s moduli of interstitial Fe-C alloys: (**a**) FeC-0.2% and (**b**) FeC-0.4% at various temperatures. For comparison, experimental [62] and SMM-calculated data by Tinh et al. [63] are also included.

The best RF-MEAM potential was then used to reproduce the elastic properties of the disordered alloys of Fe-C at various temperatures. Young’s modulus of elasticity for FeC-0.2% and FeC-0.4% were calculated and compared with the experimental data reported by [62] and the SMM (Statistical Moment Method) calculated data reported by Tinh et al. [63]. The comparison is depicted using the graph in Figure 3. As seen in the figure, Young’s modulus decreased with increasing temperatures and closely follows the experimental curve in the case of both disordered alloys. For both alloys, our potential was able to reproduce Young’s modulus for a wide range of temperatures more accurately (i.e., closely following the experimental result) than the previously reported SMM result. Detailed results on potential verification can be found in our previous work [28].

### 3.2. Data Analysis and Feature Selection

From the MD calculation result, we extracted temperature, carbon composition, and other structural parameters, including lattice constants, base structure, interstitial site placements, alloy type (i.e., ordered or disordered), and the calculated elastic moduli. To gain insights into potential input features for our ML study, we plotted the elastic moduli against these possible input features.

**Figure 4:**
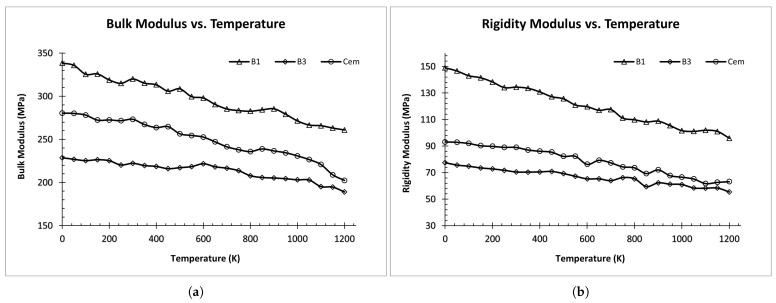
Elastic Modulus vs. Temperature for ordered structures: (**a**) Bulk Modulus vs. Temperature. (**b**) Rigidity Modulus vs. Temperature.

As shown in Figure 4, bulk and rigidity modulus tends to decrease as the temperature increases for all ordered structures (i.e., B1, B3, and Cementite). This figure also sheds light on the influence of structural features on elastic properties. However, it is not clear whether this effect is due to the lattice type or other structural parameters like lattice constants. Hence, both the lattice type and the lattice constants are taken as input features for our prediction model.

**Figure 5:**
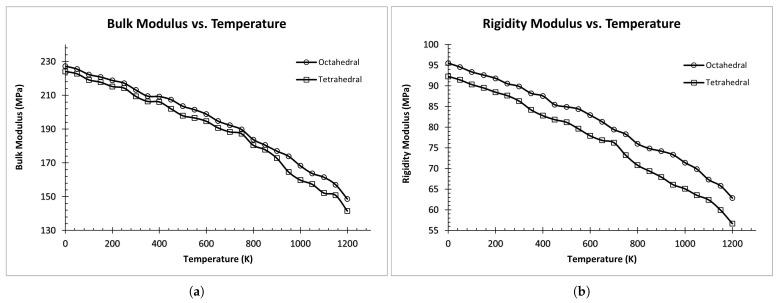
Elastic Modulus vs. Temperature for Fe-C (1% C) disordered alloy with C at different interstitial sites. (**a**) Bulk Modulus vs. Temperature, (**b**) Rigidity Modulus vs. Temperature.

Figure 5 shows the relationship between the elastic moduli and temperatures for the fcc-FeC (1% C) structure with carbon occupying octahedral and tetrahedral interstitial sites. Notably, we discerned that these properties exhibited small variations depending on the position of the carbon interstitial site. When carbon was situated at octahedral sites, both the bulk and rigidity modulus exhibited higher values than when carbon occupied tetrahedral sites. Consequently, we included the carbon interstitial site locations in our pool of potential input features.

**Figure 6:**
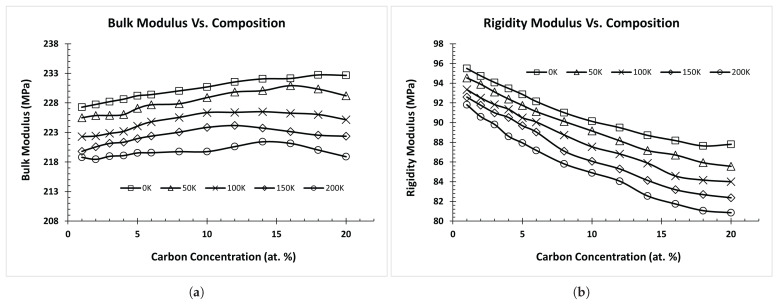
Elastic Moduli vs. Composition for various disordered Fe-C alloys with octahedral interstitial C atoms. (**a**) Bulk Modulus vs. Composition, (**b**) Rigidity Modulus vs. Composition.

Figure 6 represents the relationship between the elastic moduli and carbon composition at different temperatures for the fcc-FeC alloy. In this figure, we observed that increasing carbon composition led to an increase in the bulk modulus, up to approximately 15%, before exhibiting a gradual decrease at higher concentrations. Simultaneously, the rigidity modulus decreased with both an increase in carbon concentration and elevated temperatures. Young’s modulus is not presented in any of the three cases (Figure 4, Figure 5 and Figure 6), as it exhibited a consistent and parallel pattern to the shear modulus, owing to the greater weightage of shear modulus compared to bulk modulus in Equation (7).

Based on the data analysis above, we selected eight key input features to capture the essential variables that influence the properties of our materials. These features include temperature (Temp), carbon composition (CC), alloy type (Alloy), i.e., order or disorder alloy, interstitial sites of carbon (Int. Sites), lattice structure type (Str), and lattice constants: L*_a_*, L*_b_*, and L*_c_*.

Temperature, which is a critical parameter, ranges from 0 to 1200 K, with increments of 50 K, providing a comprehensive range of temperature conditions to evaluate material behavior. Carbon composition is another vital input, varying from 1 to 20% for disordered alloys and up to 50% for ordered alloys, encompassing a wide spectrum of compositions relevant to our study. Another important input feature is lattice type which covers four major lattice types: FCC, BCC, orthorhombic, and simple cubic, ensuring that we account for a diverse set of structural configurations. Additionally, we have included interstitial sites of carbon as a feature, with variations in octahedral, tetrahedral, planar, and basal interstitial sites. This inclusion allows us to explore the impact of carbon placement within the lattice on material characteristics.

**Figure 7:**
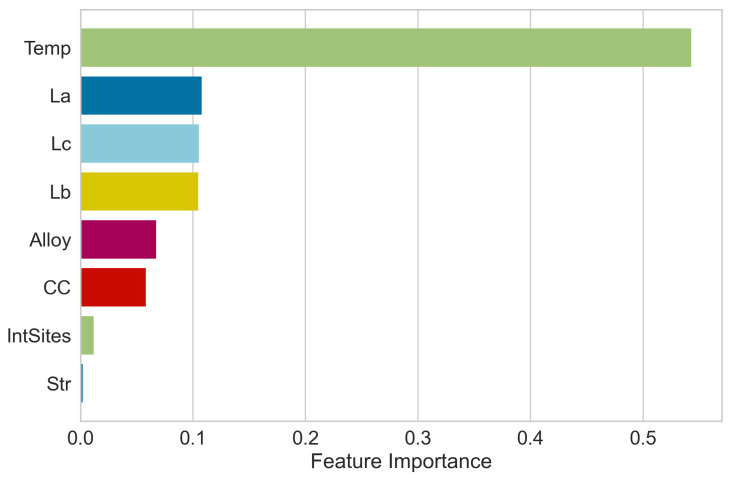
Feature Importance of eight input features for elastic properties prediction.

Next, feature analysis was performed using the ML algorithm, and RF regressor, where feature importance scores were calculated for each input feature as shown in Figure 7. Upon calculating the feature importance of the eight features against the target output, it was observed that temperature (Temp) was the most important feature, with the feature important score of 0.52. Following Temp, lattice constants (L*_c_*, L*_a_*, and L*_b_*), type of alloy (Alloy), carbon concentration (CC), and interstitial sites (IntSites) were also found to be significant. Lattice structure (Str) had a feature importance score of less than 1%, which shows that it has less impact on the target output.

The interatomic potential used to perform the MD calculation was developed on the data of just two structures of Fe-C, i.e., B1 and Cementite. So, the result obtained from MD calculation using such potential might not be sensitive to the lattice structure type. This might have resulted in the lower feature importance of lattice structure (Str) while predicting the elastic moduli.

### 3.3. Feature Elimination and Hyperparameter Tuning

Before making decisions about eliminating features based on their importance scores, we conducted another test to understand how the removal of less important features would impact the predictive accuracy of our ML models. Hence, we prepared two different datasets, one with seven features and another with all (eight features). As discussed in the “Data and Method” section, various algorithms were used on these datasets to make the prediction. Furthermore, two different approaches were tried: (a) training two independent models to predict B and G, and (b) training a multi-variate model to predict both B and G simultaneously. For all cases, the data were split into 80:20 training and test sets. The training set was used to train the models, and the test set was used to assess the models’ accuracy.

In parallel, we also performed hyperparameter tuning, which involves exploring various hyperparameters for each algorithm and selecting the most optimum solution based on the cross-validation (CV) score. Five fold CV was applied on the training set. First, individual algorithms were tuned for each dataset and method. Subsequently, for SL, the hyperparameter-tuned individual algorithms were taken as the base predictors, and at the same time, all of these were tried one by one for meta learners while tuning the hyperparameters of the meta estimator. Among all the individual algorithms tried, MLP gave the least mean square error CV score, thus was finalized as the meta estimator.

Table 2 presents the results of our experiments, showcasing the best CV scores, measured in terms of mean squared error (MSE), for each of the cases. The table shows a consistent pattern across all algorithms and models: the dataset with seven parameters consistently yields the same or superior performance compared to the dataset with eight parameters. It can be deduced that the eighth parameter, i.e., lattice structure (L) produces a neutral or negative impact on the prediction. Hence, the feature was removed from the dataset, and the results hereafter use the seven-feature dataset.

**Figure 8:**
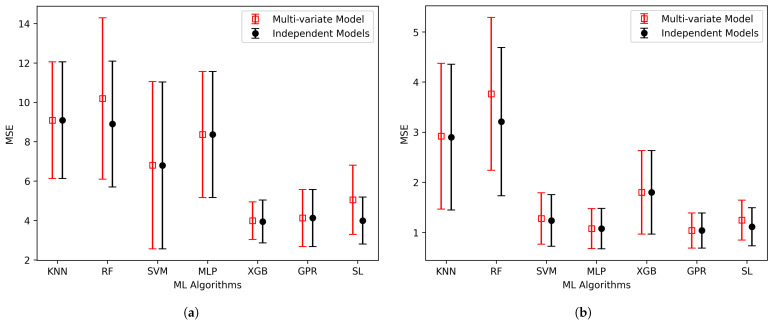
Assesment of elastic moduli, (**a**) Bulk modulus and (**b**) Rigidity modulus, calculated by independent and simultaneous models using various algorithms.

We also did a comparative analysis of two distinct approaches: (a) employing individual models for the separate prediction of the elastic constants B and G, and (b) employing a multi-variate model to predict these properties simultaneously. Figure 8 provides a comprehensive overview of this comparison, utilizing six individual models and one ensemble algorithm, focusing on the MSE as the evaluation metric. These MSE values, unlike the one presented in Table 2, are calculated using the test dataset, which has not been used during the model training. Notably, the results shown in the figure clearly favor the independent models, which consistently had smaller MSEs across all algorithms. This highlights the effectiveness of the independent model when contrasted with the multi-variate model approach.

**Figure 9:**
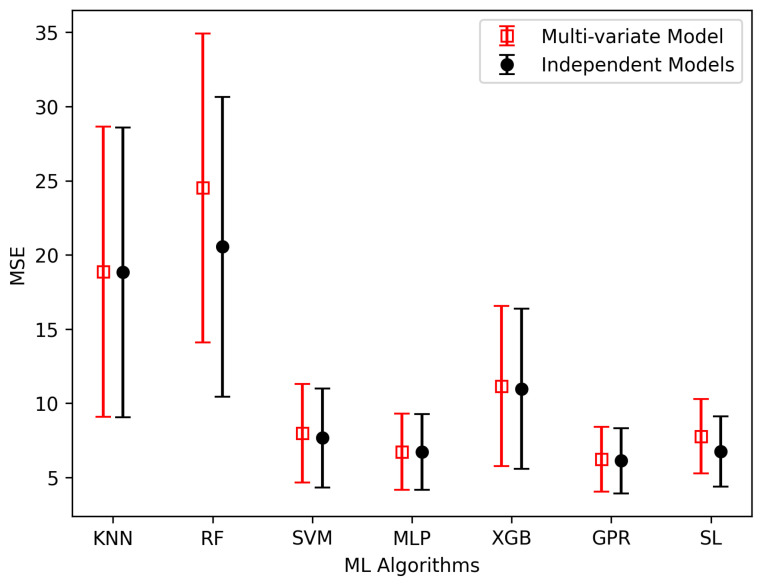
Assesment of Youngs modulus calculated by independent and simultaneous models using various algorithms.

We then also calculated Young’s modulus of elasticity (E) utilizing Equation (7), and subjected it to a similar comparative analysis, as illustrated in Figure 9. Once again, the independent model demonstrated superior performance, showcasing each algorithm’s lowest MSE when predicting Young’s modulus.

Furthermore, when examining the performance of each algorithm in Figure 8 and Figure 9 concerning the prediction of B, G, and E, some noteworthy trends emerged. In the case of B prediction, XGBoost, GPR, and SL consistently outperformed others, displaying both lower prediction errors and reduced error variation. Similarly, when estimating G, MLP, GPR, and SL demonstrated superior predictive accuracy, with GPR standing out as the top performer.

Likewise, the prediction of E, MLP, GPR, and SL proved to be the most effective, with GPR being the most accurate result. Notably, in both Figure 8 and Figure 9, we can also see that GPR and SL have been consistently effective in predicting all elastic moduli, exhibiting a narrow range of error variation and reinforcing their reliability in our analysis. The MSE errors and 95% confidence interval (CI) shown in Figure 8 and Figure 9 are also detailed in Table 3.

**Figure 10:**
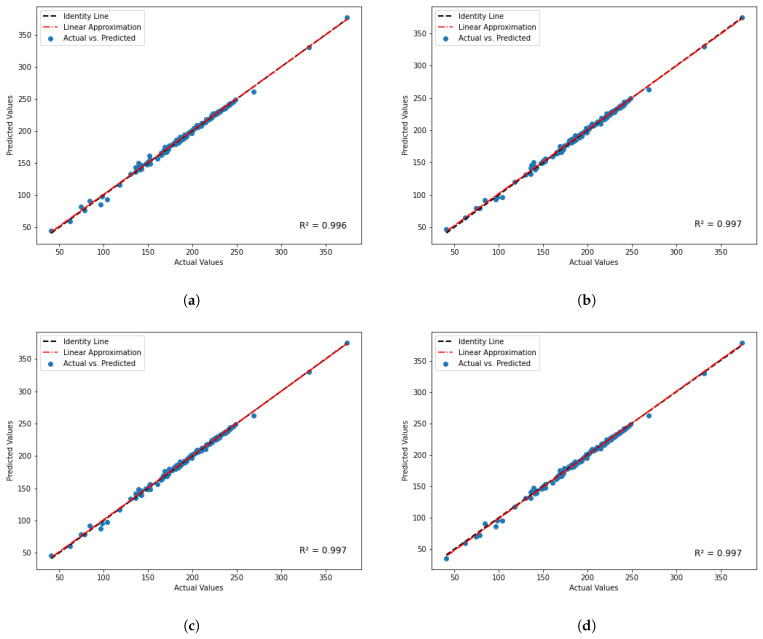
Predicted value versus true value using simultaneous B and G predicting model for top four algorithms: (**a**) SVM, (**b**) MLP (**c**) GPR, and (**d**) SL.

Now, focusing on the independent model approach for predicting B and G, we proceeded with the predictions and subsequently calculated E using Equation (7). For the ease of comparing different algorithms, the calculated Young’s modulus was compared with its actual counterpart since it will give the aggregate performance of two independent models. Figure 10 provides a visual representation of these predicted versus actual value comparisons, complete with their respective R^2^ scores. Among the seven algorithms used, four algorithms, namely (**a**) SVM, (**b**) MLP, (**c**) GPR, and (**d**) SL, showed exceptional performance with R^2^ scores of 0.996, 0.997, 0.997, and 0.997, respectively. It is particularly worth highlighting that, once again, GPR and SL demonstrated superior performance, reaffirming their effectiveness in this elastic properties prediction model.

## 4. Conclusions

In conclusion, the RF-MEAM potential, developed by fitting DFT-generated forces, energies, and stress tensors, was used to generate thousands of data points for ML, focused on predicting the elastic moduli (i.e., bulk modulus (B), rigidity modulus (G), and Young’s modulus (E)) for various Fe-C alloys. We explored a broad spectrum of ordered and disordered alloys, each characterized by varying carbon compositions, and across a range of elevated temperatures. In our quest to identify key input features for our ML models, we conducted meticulous data analysis with an in-depth understanding of the input-output relationships. This process led us to select eight input features: Temp, CC, IntSites, Alloy, Str, L*_a_*, L*_b_*, and L*_c_*. RF regressor was used to assess the importance of these features. Temp was the most influential feature, closely followed by lattice constants and alloy type. Feature importance followed by the CV accuracy evaluation during hyperparameter optimization suggested removing the least important feature, lattice structure type (Str).

Furthermore, we investigated two distinct model training approaches: independent models for B and G prediction and a multi-variate for concurrent B and G prediction. The independent models approach consistently yielded smaller MSEs across all algorithms, compellingly endorsing its effectiveness. The predictive capabilities of the independent model were further affirmed through the accurate prediction of Young’s modulus.

A thorough analysis of six individual algorithms and one ensemble algorithm was conducted, with the primary focus on comparing their performance using the R^2^ metric. Notably, the independent model approach for B and G prediction showcased remarkable results, particularly for GPR and SL, both achieving remarkable R^2^ scores of 0.997 when predicting E. This outcome further solidifies their standing as robust and dependable predictors in the context of our study.

Our study has thus contributed valuable insights into the ML prediction of elastic properties for Fe-C alloys, emphasizing the significance of temperature and a select set of input features. It underscores the effectiveness of the independent model approach and the robust performance of GPR and SL, paving the way for enhanced material design and optimization in materials science.

## Figures and Tables

**Table 1:** Comparison of our results with the published DFT data for equilibrium volume, bulk modulus, and pressure derivative calculated by the Birch–Murnaghan equation of state.

Structure	V_0_ (Å^3^)		B_0_ (GPa)		B_0_′	
	**RF-** **MEAM**	**Literature**	**RF-** **MEAM**	**Literature**	**RF-** **MEAM**	**Literature**
B1 (FeC)	65	64 ^a,b^	338	329 ^a^	5.28	4.40 ^a^
Cementite (Fe_3_C)	155	154 ^a^	252	234 ^a^	4.05	4.00 ^a^

^a^ Henriksson et al. [59]; ^b^ Chihi et al. [60].

**Table 2:** Best CV scores across different ML models and feature sets during hyperparameter tuning.

Algorithms	Independent Models				Multi-Variate Model	
	**B_7**	**B_8**	**G_7**	**G_8**	**S_7**	**S_8**
RF	8.311	8.377	3.878	3.885	7.046	7.050
KNN	11.051	11.051	3.447	3.447	7.250	7.250
SVM	8.427	8.427	1.646	1.646	10.175	10.175
MLP	5.470	5.485	1.514	1.616	3.520	3.596
GPR	5.036	5.036	1.453	1.453	3.244	3.244
XGBoost	4.868	4.868	1.517	1.517	3.259	3.259
SL	3.876	4.695	1.360	1.441	3.440	3.696

**Table 3:** Comparison for MSE calculated on test set for multi-variate and independent models using different ML algorithms.

Algorithms	B		G		E	
	**Multi-Variate**	**Independent**	**Multi-Variate**	**Independent**	**Multi-Variate**	**Independent**
KNN	9.09 ± 2.97	9.09 ± 2.97	2.92 ± 1.46	2.90 ± 1.46	18.86 ± 9.81	18.83 ± 9.81
RF	10.20 ± 4.11	8.90 ± 3.21	3.76 ± 1.53	3.21 ± 1.48	24.52 ± 10.45	20.55 ± 10.14
SVM	6.81 ± 4.26	6.80 ± 4.25	1.28 ± 0.52	1.24 ± 0.51	7.96 ± 3.35	7.66 ± 3.33
MLP	8.36 ± 3.21	8.36 ± 3.21	1.08 ± 0.40	1.08 ± 0.40	6.72 ± 2.59	6.71 ± 2.56
XGB	3.99 ± 0.96	3.95 ± 1.09	1.80 ± 0.83	1.80 ± 0.83	11.15 ± 5.42	10.97 ± 5.41
GPR	4.13 ± 1.45	4.13 ± 1.45	1.04 ± 0.35	1.04 ± 0.35	6.32 ± 2.21	6.22 ± 2.20
SL	5.05 ± 1.76	4.00 ± 1.20	1.25 ± 0.40	1.11 ± 0.38	7.77 ± 2.53	6.75 ± 2.38

## Data Availability

The data presented in this study are available on request from the corresponding authors.
